# A Clofazimine-Containing Regimen Confers Improved Treatment Outcomes in Macrophages and in a Murine Model of Chronic Progressive Pulmonary Infection Caused by the *Mycobacterium avium* Complex

**DOI:** 10.3389/fmicb.2020.626216

**Published:** 2021-01-14

**Authors:** Ju Mi Lee, Jiyun Park, Sangwon Choi, Byung Woo Jhun, Su-Young Kim, Kyung-Wook Jo, Jung Joo Hong, Lee-Han Kim, Sung Jae Shin

**Affiliations:** ^1^Department of Microbiology, Institute for Immunology and Immunological Disease, Brain Korea 21 Program for Leading Universities and Students (PLUS) Project for Medical Science, Yonsei University College of Medicine, Seoul, South Korea; ^2^Division of Pulmonary and Critical Care Medicine, Department of Medicine, Samsung Medical Center, Sungkyunkwan University School of Medicine, Seoul, South Korea; ^3^Division of Pulmonology and Critical Care Medicine, Department of Internal Medicine, Asan Medical Center, University of Ulsan College of Medicine, Seoul, South Korea; ^4^National Primate Research Center, Korea Research Institute of Bioscience and Biotechnology, Cheongju, South Korea

**Keywords:** *Mycobacterium avium* complex-pulmonary disease, clofazimine, clofazimine-containing regimen, standard treatment regimen, minimum inhibitory concentrations, intracellular drug susceptibility test, *in vivo* drug susceptibility test, chronic progressive murine model

## Abstract

Treatment outcomes using the standard regimen (a macrolide, ethambutol, and rifampicin) for *Mycobacterium avium* complex-pulmonary disease (MAC-PD) remain unsatisfactory. Thus, improved treatment regimens for MAC-PD are required. Clofazimine has recently been revisited as an effective drug against mycobacterial infection. We performed a comparison between the standard regimen and an alternative regimen (replacing the rifampicin of the standard regimen with clofazimine) based on the intracellular anti-MAC activities of the individual drugs in a murine model of chronic progressive MAC-pulmonary infection (MAC-PI). The intracellular anti-MAC activities of the individual drugs and their combinations in murine bone marrow-derived macrophages (BMDMs) were determined. The treatment efficacies of the standard and clofazimine-containing regimens were evaluated in mice chronically infected with *M. avium* by initiating 2- and 4-week treatment at 8 weeks post-infection. Bacterial loads in the lung, spleen, and liver were assessed along with lung inflammation. Insufficient intracellular anti-MAC activity of rifampicin in BMDMs was recorded despite its low *in vitro* minimum inhibitory concentrations (MICs), whereas optimal intracellular killing activity against all tested MAC strains was achieved with clofazimine. Compared to the standard regimen, the clofazimine-containing regimen significantly reduced CFUs in all organs and achieved marked reductions in lung inflammation. The replacement of rifampicin with clofazimine in the treatment regimen resulted in more favorable outcomes in an animal model of chronic progressive MAC-PI. Intriguingly, 2 weeks of treatment with the clofazimine-containing regimen reduced bacterial loads more effectively than 4 weeks of treatment with the standard regimen in *M. avium*-infected mice. Thus, the clofazimine-containing regimen also had a treatment-shortening effect.

## Introduction

Non-tuberculous mycobacteria (NTM), which are ubiquitous and opportunistic pathogens, comprise all the members of *Mycobacterium* except the *Mycobacterium tuberculosis* complex and *Mycobacterium leprae* (Baldwin et al., 2019; Daley et al., 2020). The incidence and prevalence of infectious disease caused by NTM have been increasing globally, leading to an emerging problem related to the difficulty of treatment (Loebinger, 2017; Lee et al., 2019).

The *Mycobacterium avium* complex (MAC), which comprises slow-growing *Mycobacterium* species, has been ranked as the most common group of NTM pathogens worldwide (Hoefsloot et al., 2013; Daley et al., 2020). In particular, *M. avium* and *Mycobacterium intracellulare* are the main species causing pulmonary disease (PD) and include two major forms: nodular bronchiectatic and fibrocavitary (Koh, 2017). Patients with drug-susceptible MAC-PD generally receive at least three antibiotics, a macrolide [azithromycin or clarithromycin (CLR)], ethambutol (EMB), and rifampicin (RIF), as a standard regimen for at least 12 months based on previous empirical clinical investigations (Griffith et al., 1996; Wallace et al., 1996; Wallace et al., 2014; Griffith, 2018). Although the clinical effectiveness of EMB and RIF in MAC-PD treatment has not been fully verified, these drugs are used as supportive treatments to prevent microbiological resistance to macrolide monotherapy (Kim et al., 2019). In addition, the minimum inhibitory concentrations (MICs) of EMB and RIF are associated with treatment efficacy, and 85.3% of patients whose MAC isolates have MICs of <8 mg/L for RIF and/or EMB have successful treatment (Kwon et al., 2018).

However, the serum concentrations of macrolides, the key drugs for the treatment of MAC disease, have been reported to be low because of the interplay with RIF (Koh et al., 2012; Shimomura et al., 2015; Jeong et al., 2016). RIF is a potent inducer of cytochrome P450 (CYP3A) enzymatic activity, and macrolides are CYP3A inhibitors. However, due to the prominent effect of RIF-mediated CYP3A activation, coadministration of RIF and a macrolide may lead to poor treatment outcomes (Shimomura et al., 2015; Akiyama et al., 2019). Moreover, increased serum concentrations of RIF can cause side effects, such as liver damage, leukopenia, and thrombocytopenia (Shimomura et al., 2015). In addition, a previous study revealed that a treatment regimen consisting only of a macrolide and EMB without RIF resulted in non-inferior outcomes compared with those of the standard three-drug regimen in selected cases (Miwa et al., 2014). Furthermore, Jarand et al. (2016) reported that patients treated with a macrolide, EMB, and clofazimine (CFZ) instead of RIF showed better treatment outcomes than those treated with a RIF-containing regimen. All these results have raised the question of whether RIF plays a sufficient role in MAC therapy despite the low *in vitro* MIC value of RIF against MAC strains.

CFZ has been used mainly to treat drug-resistant tuberculosis and leprosy (Tyagi et al., 2015). Moreover, it has been introduced for the treatment of NTM-PD caused by not only MAC but also *Mycobacterium abscessus* (MAB), with promising results (Roussel and Igual, 1998; Field and Cowie, 2003; Jarand et al., 2016; Yang et al., 2017). Particularly, in the treatment of MAC-PD, the CFZ-containing regimen showed favorable treatment outcomes in treatment-naïve patients as well as those with refractory disease (Jarand et al., 2016; Martiniano et al., 2017). In addition, under the guidelines from the Clinical and Laboratory Standards Institute, interest in combinatory regimens containing CFZ has re-emerged, even for the treatment of macrolide-resistant MAC infections (CLSI, 2018). Notably, CFZ had a bacteriostatic effect, and its use in combination with CLR had a synergistic ability to prevent regrowth of MAB and MAC *in vitro* (Ferro et al., 2016).

However, the clinical use of CFZ based on drug susceptibility testing (DST) still lacks sufficient evidence due to the unclear and unreliable breakpoint for CFZ susceptibility testing (Huang et al., 2018; Luo et al., 2018). Additionally, correlations between the results from *in vitro* DST, intracellular activity, and *in vivo* treatment outcomes have not been clearly investigated for MAC clinical isolates (Huang et al., 2018). Although a synergistic effect of CLR with CFZ was demonstrated in a murine model of *M. avium* ATCC 700898 infection (Lanoix et al., 2020), our present study, to the best of our knowledge, is the first to evaluate CFZ activity against a chronic and progressive infection in a murine model that reflects the clinical situation, along with an investigation of its intracellular anti-MAC activity. Generally, MAC-PD treatment is initiated once disease progression is noted radiologically or once symptoms worsen in patients who have chronic MAC lung infection, with significant bacterial loads and severe inflammation (Thomson and Yew, 2009). Thus, the establishment of chronic MAC-PI in animal models to mimic the condition of patients is necessary to rationally devise more effective MAC-PD chemotherapy regimens. However, the main limitation of *in vivo* studies is that chronic infection with MAC strains via aerosolization is not easily achieved in mice. Accordingly, the majority of experimental studies of MAC infection *in vivo* have employed intraperitoneal, intratracheal or intranasal injection rather than aerosol infection (Amaral et al., 2011; Bruffaerts et al., 2017; Abdissa et al., 2018; Kannan et al., 2019). Our previous study mimicked chronic infection with different MAC strains via aerosolization to investigate the possibility of reactivation of MAC infection, and it revealed that the different MAC strains in the mice exhibited different growth rates, which might be associated with differences in host-pathogen interactions (Cha et al., 2015).

In the present study, to compare efficacy between these regimens in MAC-infected mice, we used *M. avium* SMC #7, a clinical isolate, to cause a chronic progressive pulmonary infection (PI) in mice. The main purpose of this study was to evaluate the efficacy of the standard regimen and an alternative regimen that replaced RIF with CFZ in a murine model of chronic progressive MAC infection based on the *in vitro* and intracellular activities of individual drugs against MAC clinical isolates.

## Materials and Methods

### Mycobacterial Strains and Culture Conditions

Eight *Mycobacterium* species were used in this study (Table 1). Briefly, reference strains were obtained from the American Type Culture Collection (ATCC; Manassas, VA, United States), and all clinical isolates were obtained from patients with the typical nodular bronchiectatic form of MAC-PD who had never been exposed to antimycobacterial antibiotics at the time of diagnosis (Samsung Medical Center, SMC; Seoul, South Korea). All strains were cultivated in Middlebrook 7H9 broth (BD-Difco, Pont-de-Claix, France) supplemented with 10% oleic acid-albumin-dextrose-catalase (OADC) at 37°C. Single-cell suspensions of each strain were prepared as previously described (Cha et al., 2015), and colony-forming units (CFUs) were determined on Middlebrook 7H10 agar plates (BD-Difco).

**TABLE 1:** Minimum inhibitory concentrations (MICs) of anti-mycobacterial drugs against *Mycobacterium* strains used in this study.

		MIC (mg/L)			
Species	Strains	CLR	EMB	RIF	CFZ
*M. avium*					
	CP000479.1*	1	8	4	2
	ATCC 700898*	0.5	8	1	4
	SMC #1	0.5	4	0.5	4
	SMC #7	0.5	8	4	2
*M. intracellulare*					
	ATCC 13950*	0.25	4	0.25	2
	SMC #8	0.5	4	2	4
	SMC #11	1	1	0.5	1
*M. tuberculosis*					
	ATCC 27294*	*N*.*D*.	2	0.25	0.25

CLR, clarithromycin; EMB, ethambutol; RIF, rifampicin; CFZ, clofazimine; SMC, Samsung Medical Center (Seoul, South Korea); N.D., not determined. *These strains were used as controls for the relevant experiments in this study.

### Antibiotics

All drugs (CLR, EMB, RIF, and CFZ) were purchased from Sigma-Aldrich, Inc. (St. Louis, MO, United States) and were diluted in Dulbecco’s phosphate-buffered saline (DPBS; Biowest, Nuaillé, France) after complete dissolution in dimethyl sulfoxide for *in vitro* DST and intracellular activity testing. For *in vivo* oral administration, 0.5% carboxymethylcellulose was used as a vehicle.

### Ethics and the Animal Model

Specific pathogen-free female BALB/c and C57BL/6 (6–8 weeks of age) mice were purchased from Japan SLC, Inc. (Shijuoka, Japan). All animal experiments were conducted in accordance with the Korean Food and Drug Administration (KFDA) guidelines and approved by the Ethics Committee and Institutional Animal Care and Use Committee of the Laboratory Animal Research Center at Yonsei University College of Medicine (Permit Number: 2015-0273 and 2018-0229).

### *In vitro* Susceptibility Testing

*In vitro* susceptibility testing of MAC strains to individual drugs was determined by the broth microdilution resazurin assay after 7 days of incubation, as previously described. The MICs of the drugs were defined as the lowest concentration of the agents that inhibited bacterial growth. All MIC results for the tested MAC strains were verified by conducting each test twice (in duplicate wells).

### Intracellular Anti-MAC Activities of Individual Drugs and Their Combinations

Murine bone marrow-derived macrophages (BMDMs) from BALB/c and C57BL/6 mice were differentiated in Dulbecco’s modified Eagle’s medium (Biowest) supplemented with 10% fetal bovine serum (Biowest) and 10% L929 supernatant as previously described (Choi et al., 2019) and used for intracellular anti-MAC activities of the tested drugs at the indicated concentrations. Briefly, BMDMs (3 × 10^5^ cells/ml) were cultured in a 48-well plate for 24 h, infected with the indicated MAC strains (Table 1) at multiplicity of infection of 3 for 4 h, and washed with DPBS. The cells were then cultured with or without drugs in duplicate or triplicate wells for 72 h. The cells were then lysed with 0.05% Triton X -100, and the lysates were serially diluted with DPBS and spotted a maximum of four times per well on 7H10 agar plates supplemented with 10% OADC to quantify the numbers of viable bacteria. Colonies were counted after 14 days of incubation at 37°C, and the values are reported as the mean CFUs ± standard deviations per ml of BMDMs. Each experiment was repeated at least twice independently.

### Head-to-Head Assessment of Treatment Efficacy Between Standard and CFZ-Containing Regimens in an Animal Model of Chronic Progressive *M. avium* Pulmonary Infection

For *in vivo* experiments, a total of 44 BALB/c mice were infected with *M. avium* SMC #7 via aerosolization as previously described (Cha et al., 2015). Briefly, the mice were exposed to *M. avium* SMC #7 via aerosolization using an inhalation exposure system (Glas-Col, Terre Haute, IN, United States). Four to five mice per group were euthanized at 0, 2, 4, and 8 weeks for the establishment phase of the infection, two and four mice in the control group were euthanized at 10 weeks and 12 weeks post infection, and every five mice in the standard regimen group and CFZ-containing regimen group were euthanized at 10 and 12 weeks for evaluation of the bacterial loads in the lung, spleen and liver and histopathological analysis of the lung. At 8 weeks post-infection, treatment with each regimen 5 days per week for 2 and 4 weeks was initiated by oral gavage. The drug doses were as follows: 100 mg/kg for CLR and EMB, 10 mg/kg for RIF and 20 mg/kg for CFZ.

Organ homogenates were plated through serial dilution onto 7H10 agar plates supplemented with 10% OADC and 0.5% amphotericin B (Sigma-Aldrich) for CFU determination. Bacterial colonies were counted after incubation at 37°C for 2 weeks. For histopathological analysis, the right superior lobes of the lungs were preserved in 10% neutral buffered formalin, embedded in paraffin and sectioned at 4–5 μm, followed by hematoxylin and eosin staining. The extent of the affected inflamed area in the lungs was analyzed using open-source ImageJ software (National Institutes of Health, Bethesda, MD, United States).

### Statistical Analysis

The Mann–Whitney test was used to evaluate differences between two groups, and ANOVA corrected with Tukey’s test was used to evaluate differences among multiple groups using GraphPad Prism version 7 (San Diego, CA, United States). A *p*-value < 0.05 was considered statistically significant.

## Results

### Determination of the *in vitro* MIC Values and Intracellular Anti-MAC Activities of Individual Antibiotics

The MICs of CLR, EMB, RIF, and CFZ for the MAC strains used in this study are given in Table 1. Overall, the MAC strains were susceptible to CLR according to Clinical and Laboratory Standards Institute breakpoints (CLSI, 2018) but exhibited varying susceptibility to EMB, RIF, and CFZ, as previously reported (Huang et al., 2018; Wetzstein et al., 2020).

**FIGURE 1:**
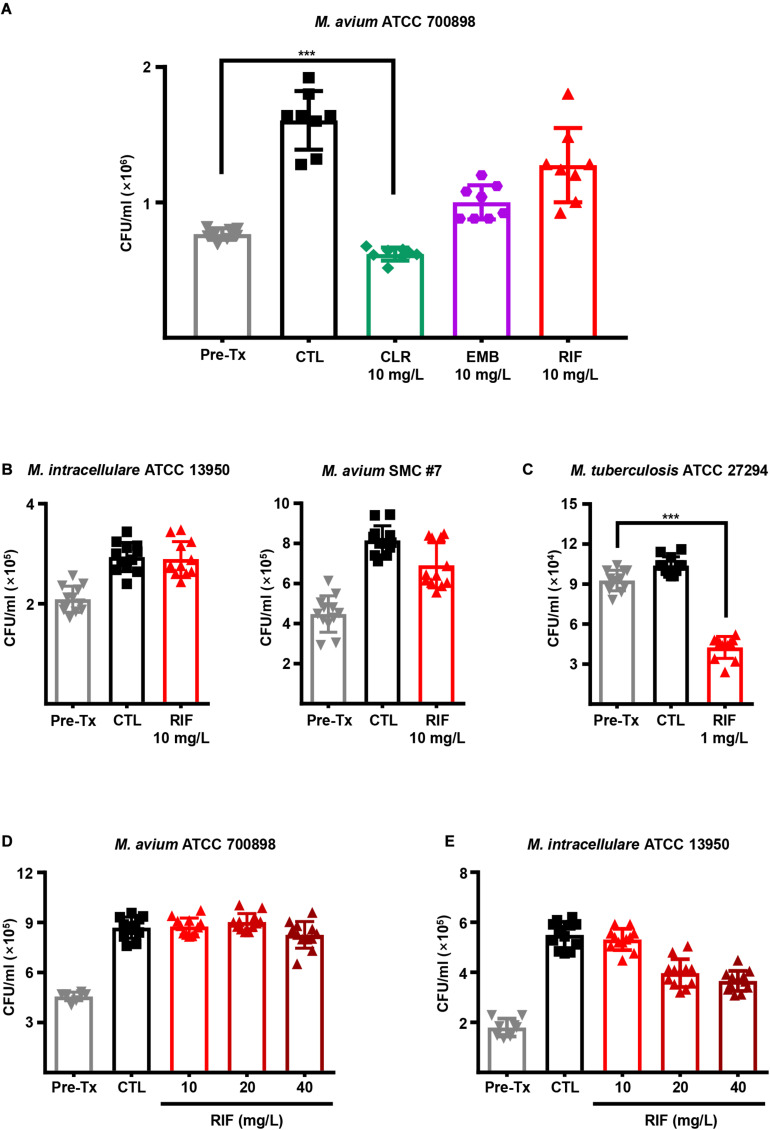
Intracellular anti-MAC activities of first-line drugs in MAC-infected BMDMs. **(A)** BMDMs were infected with *M. avium* ATCC 700898 and treated with the indicated doses of CLR, EMB, and RIF. Unless otherwise indicated, all experiments investigating the intracellular activities of drugs in BMDMs were evaluated at 72 h post-infection by plating serially diluted cell lysates onto 7H10-OADC agar plates. **(B)** BMDMs were infected with *M. intracellulare* ATCC 13950 or *M. avium* SMC #7 and treated with 10 mg/L RIF. **(C)** BMDMs were infected with *M. tuberculosis* H37Rv and treated with 1 mg/L RIF as a control experiment. BMDMs were infected with **(D)** *M. avium* ATCC 700898 or **(E)** *M. intracellulare* ATCC 13950 and treated with the indicated doses of RIF. Each experiment was repeated at least twice independently with duplicate or triplicate wells; the results of a representative experiment are shown. Each dot represents the mean value ± S.D. of duplicate or triplicate wells, with four spots applied per well. The Mann–Whitney test was used to evaluate significance, and the results are represented as the mean value ± S.D. ***p < 0.001 vs. Pre-Tx. Pre-Tx, pre-treatment; CTL, untreated control.

Next, we evaluated the intracellular activities of first-line antibiotics to determine whether a high dose of each drug is effective in inhibiting MAC growth inside macrophages. BMDMs from BALB/c mice were infected with *M. avium* ATCC 700898 and treated with 10 mg/L CLR, EMB, and RIF for 3 days (Figure 1A). Interestingly, a low MIC (1 mg/L) value was observed for RIF *in vitro* (Table 1), but RIF was insufficiently effective against *M. avium* ATCC 700898 at even a 10-fold higher concentration (10 mg/L) in BMDMs (Figure 1A). Furthermore, ineffective intracellular activity of RIF against *M. intracellulare* ATCC 13950 and *M. avium* SMC #7 in BMDMs were confirmed despite its low *in vitro* MICs (0.25 and 4 mg/L, respectively), whereas significant intracellular activity of RIF against *M. tuberculosis* ATCC 27294 was observed at only 1 mg/L (Figures 1B,C). In addition, *M. avium* ATCC 700898-infected BMDMs were treated with various concentrations of first-line antibiotics. The results revealed that compared to control treatment, RIF alone had no anti-MAC activity at any concentration in BMDMs (Figure 1D and Supplementary Figure 1). Similarly, *M. intracellulare* ATCC 13950 exhibited tolerance to high RIF concentrations, even at 40 mg/L, in BMDMs (Figure 1E). Next, to investigate whether the lack of intracellular anti-MAC activity of RIF was due to specific characteristics of macrophages from BALB/c mice, BMDMs from C57BL/6 mice were infected with *M. avium* ATCC 700898, *M. avium* 104 (CP000479.1) and *M. intracellulare* ATCC 13950 and treated with 10 mg/L RIF. Interestingly, RIF was again ineffective against those MAC strains, which was similar to the results obtained for BMDMs from BALB/c mice (Supplementary Figure 2A). Similar to *M. avium* ATCC 700898, *M. avium* 104 (CP000479.1) was inhibited by CLR concentrations over 10 mg/L but tolerated a high concentration of RIF (Supplementary Figures 2B,C). Taken together, these results show that the relatively low or absent intracellular anti-MAC activity of RIF did not result from its concentration or specific characteristics of different types of BMDMs, indicating the existence of inconsistent results between *in vitro* and intracellular activities.

### Intracellular Anti-MAC Activity of CFZ

**FIGURE 2:**
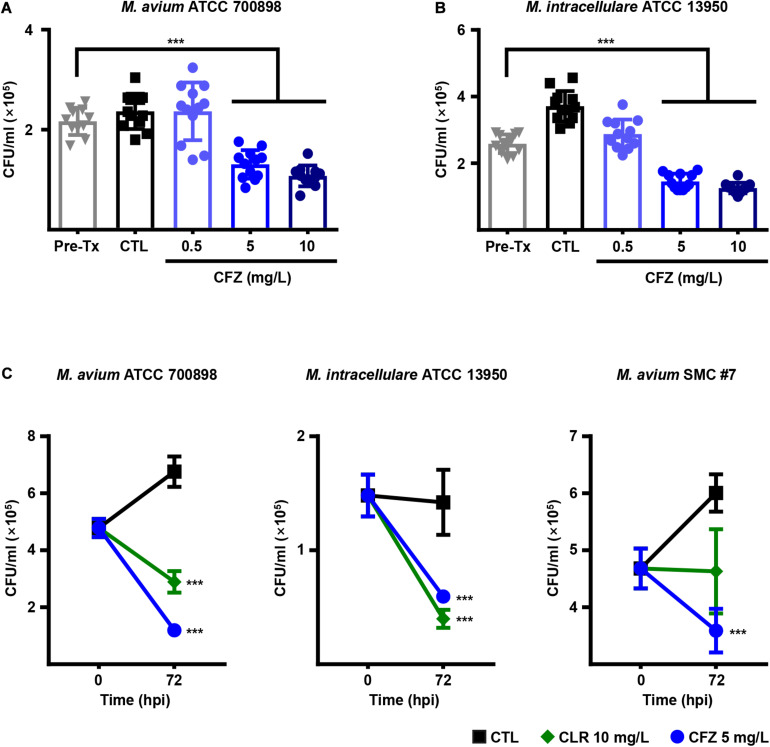
Concentration-dependent intracellular activities of CFZ in MAC-infected BMDMs. BMDMs were infected with **(A)** *M. avium* ATCC 700898 or **(B)** *M. intracellulare* ATCC 13950 and treated with the indicated dose of CFZ. **(C)** BMDMs were infected with *M. avium* ATCC 700898, *M. intracellulare* ATCC 13950 or *M. avium* SMC #7 and treated with 5 mg/L CFZ or 10 mg/L CLR. After 72 h of cultivation, bacterial CFUs were enumerated by plating serially diluted cell lysates on 7H10-OADC agar plates. Each experiment was repeated at least twice independently with triplicate wells; the results of a representative experiment are shown. Each dot represents the mean value ± S.D. of duplicate or triplicate wells, with four spots applied per well. The Mann–Whitney test was used to evaluate significance, and the results are represented as the mean value ± S.D. ***p < 0.001 vs. Pre-Tx. Pre-Tx, pre-treatment; CTL, untreated control; hpi, h post-infection.

To investigate the intracellular anti-MAC activity of CFZ in BMDMs, BMDMs were infected with *M. avium* ATCC 700898 and *M. intracellulare* ATCC 13950 and treated with different concentrations of CFZ (Figures 2A,B). Concentrations of CFZ over 5 mg/L exhibited significant intracellular activity against both strains at 72 h post-infection in BMDMs despite the relatively high MIC values for *M. avium* ATCC 700898 and *M. intracellulare* ATCC 13950 compared to those for RIF and CLR (Figure 2A,B). Next, BMDMs were infected with *M. avium* ATCC 700898, *M. intracellulare* ATCC 13950 or *M. avium* SMC #7 and treated with 5 mg/L CFZ or 10 mg/L CLR, and intracellular activity was assessed at 72 h post-infection (Figure 2C). Remarkably, CFZ inhibited intracellular bacterial growth to a similar extent as CLR.

### Comparison of Anti-MAC Activities Between the Standard and CFZ-Containing Regimens in Macrophages

**FIGURE 3:**
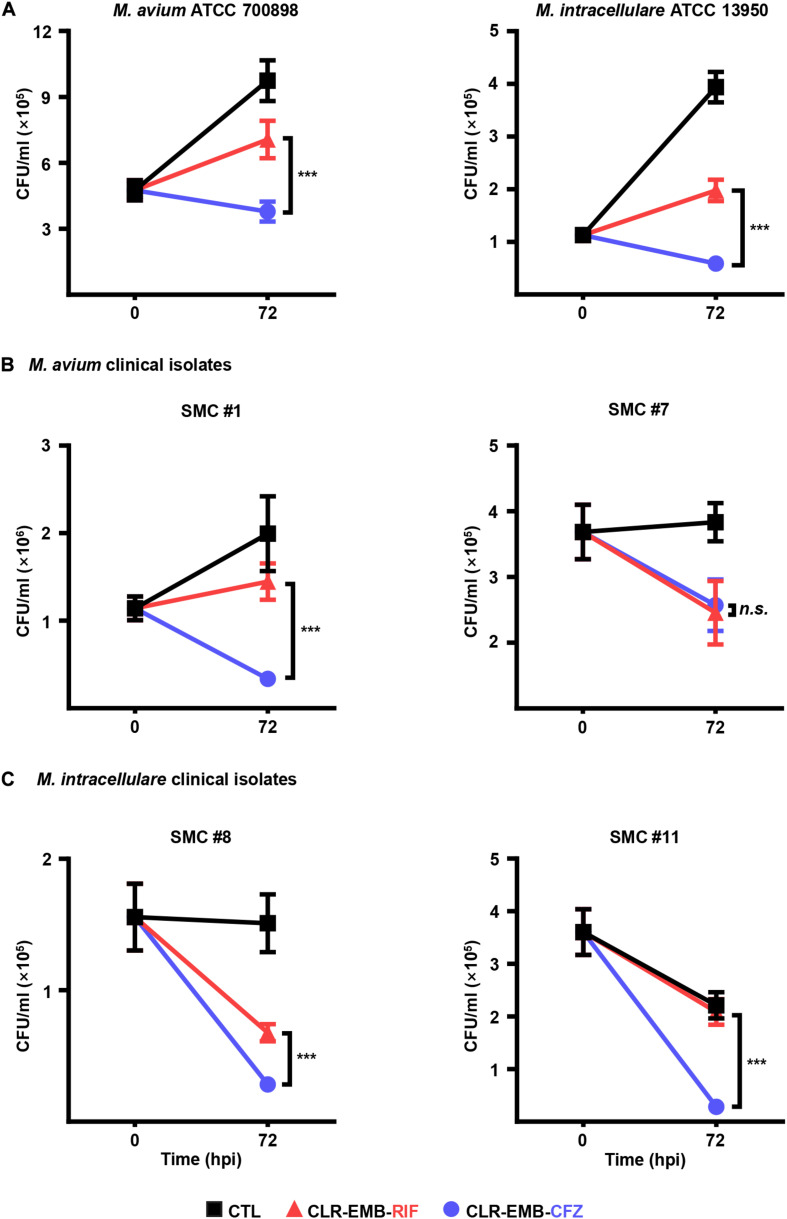
Comparative evaluation of the intracellular activities of drug combinations for the standard regimen and for the CFZ-containing regimen against a variety of MAC strains in BMDMs. BMDMs were infected with each MAC strain and treated with the drug regimen at 1 × MIC according to the data in Table 1. **(A)** *M. avium* ATCC 700898 and *M. intracellulare* ATCC 13950, **(B)** *M. avium* SMC #1 and *M. avium* SMC #7 and **(C)** *M. intracellulare* SMC #8 and *M. intracellulare* SMC #11 were assessed at 72 h post-infection by plating serially diluted cell lysates on 7H10-OADC agar plates. Each experiment was repeated at least twice independently with triplicate wells; the results of a representative experiment are shown. The Mann–Whitney test was used to evaluate significance, and the results are represented as the mean value ± S.D. ***p < 0.001 and *n.s.*, not significant. CTL, untreated control; CLR-EMB-RIF, standard regimen; CLR-EMB-CFZ, CFZ-containing regimen; hpi, h post-infection.

We next investigated the intracellular anti-MAC activities of each drug at different concentrations based on the *in vitro* MICs in BMDMs (Supplementary Figure 3). According to the *in vitro* MIC results (Table 1), the concentrations of all drugs except CFZ for monotherapy were fixed at 5 × MIC, and CFZ was fixed at 2 × MIC. The concentrations for the combined therapy were chosen to be 1 × MIC and 3 × MIC for each strain. CFZ exhibited an equally significant reduction in the viability of six MAC strains in BMDMs at 72 h post-infection (*p* < 0.001), even at only 2 × MIC. Finally, the intracellular anti-MAC activities of the standard regimen (CLR-EMB-RIF) versus the CFZ-containing regimen, as shown in Figure 3 and Supplementary Figure 4, were compared. The majority of MAC strains (i.e., all strains except *M. avium* SMC #7) displayed significantly lower viability at both 1 × MIC and 3 × MIC for the CFZ-containing regimen than for the standard regimen at 72 h in BMDMs (*p* < 0.001) (Figure 3 and Supplementary Figure 4). Interestingly, bacterial growth of *M. intracellulare* SMC #11 was naturally inhibited in BMDMs without anti-MAC drugs. However, we confirmed that it was not due to cytotoxic effect of *M. intracellulare* SMC #11 on BMDMs (data not shown). Taken together, these results suggest that a concentration of 1 × MIC was sufficient to control intracellular growth (Figure 3).

### Head-to-Head Comparison of Treatment Efficacy Between the Standard and CFZ-Containing Regimens in a Murine Model of Chronic Progressive MAC-PI

**FIGURE 4:**
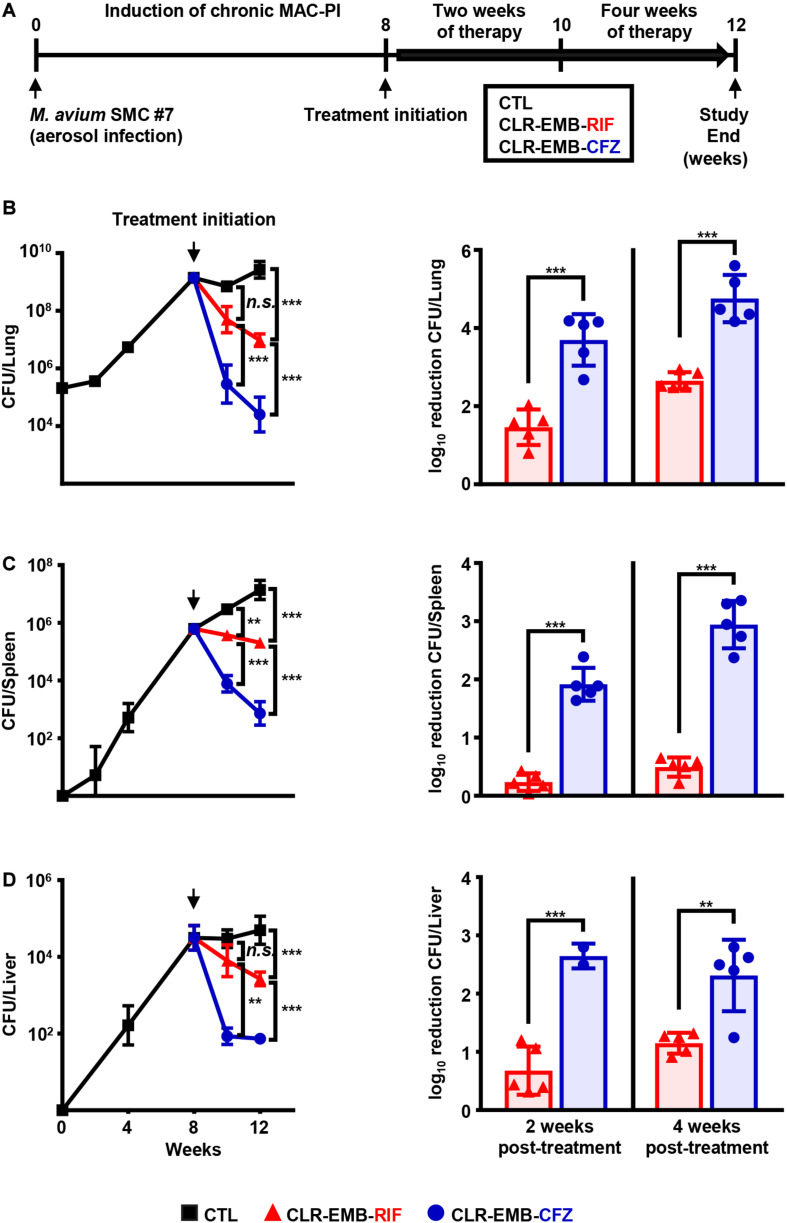
Comparative assessment of treatment efficacy of the standard and CFZ-containing regimens in a murine model of chronic progressive MAC-PI. **(A)** Schematic design of the *in vivo* experiment. BALB/c mice were infected with the *M. avium* SMC #7 clinical isolate via aerosolization, achieving a mean initial bacterial number of 2 × 10^5^ CFUs in the lungs. Two weeks and 4 weeks of therapy were initiated with the standard and CFZ-containing regimens at 8 weeks post-infection. The daily drug doses were 100 mg/kg for CLR and EMB, 10 mg/kg for RIF and 20 mg/kg for CFZ. The mice were euthanized, and the lungs were homogenized for histopathological examination and bacterial counts at 2 and 4 weeks post-treatment. Plotted infection data and mean CFU counts and bar graphs for the log_10_ reduction in CFU from treatment initiation in the **(B)** lungs, **(C)** spleens and **(D)** livers were assessed after 2 and 4 weeks of treatment with the two regimens by plating serially diluted tissue lysates onto 7H10-OADC agar plates. The broken vertical line of each plotted data indicates the day of treatment initiation. The statistical significance in **(B–D)** was calculated by one-way ANOVA followed by Tukey’s multiple comparison test, and the results are represented as the mean value ± S.D. **p < 0.01, ***p < 0.001 and *n.s.*, not significant. MAC-PI, *M. avium* complex-pulmonary infection; CTL, untreated control; CLR-EMB-RIF, standard regimen; CLR-EMB-CFZ, CFZ-containing regimen.

Since treatment for patients with MAC-PD is initiated after disease progression becomes imminent, we established a murine model of chronic progressive MAC-PI using BALB/c mice by aerosol infection with *M. avium* SMC #7 (Figure 4A). At 8 weeks post-infection, the treatment efficacies of the regimen containing CFZ instead of RIF and the standard regimen were investigated for 2 and 4 weeks (Figure 4A). The impact of the CFZ-containing regimen on bacterial load is shown as the mean and log_10_ reduction in CFU. At both 2 and 4 weeks post-treatment, there was a significant reduction in the numbers of *M. avium* SMC #7 in the CFZ-containing regimen group, with a reduction of 2.24 log_10_ units at 2 weeks and 2.10 log_10_ units at 4 weeks compared with those in the standard regimen group (*p* < 0.001). Interestingly, the bacterial load of the CFZ-containing regimen group at 2 weeks was reduced effectively compared with that of the standard regimen group at 4 weeks, with a 1.04 log_10_ reduction (*p* < 0.05) (Figure 4B). In the spleen and liver, the antibiotic activities were similar, with a reduction in activity in the lung (Figures 4C,D). In the spleen, reductions of 1.68 log_10_ units at 2 weeks and 2.45 log_10_ units at 4 weeks were obtained for the CFZ-containing regimen group compared with the standard regimen group (*p* < 0.001). In the liver, reductions of 1.97 log_10_ units at 2 weeks (*p* < 0.001) and 1.16 log_10_ units at 4 weeks (*p* < 0.01) were obtained in the CFZ-containing regimen group compared with the standard regimen group.

**FIGURE 5:**
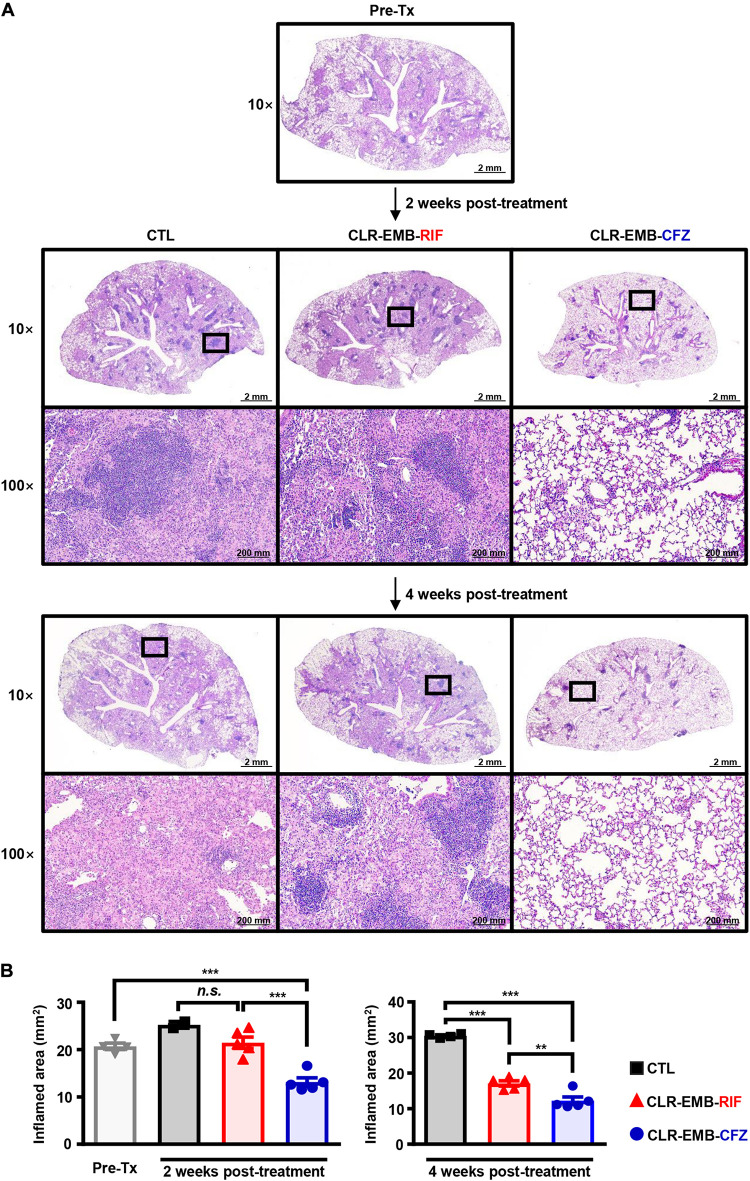
Histopathological evaluation of lung inflammation between the standard and CFZ-containing regimens at 2 and 4 weeks post-treatment. **(A)** Representative 10× and 100× magnification photomicrographs of H&E-stained lung tissue from the superior lobe of the right lung are shown. Each treatment regimen is indicated below the representative lung pathology. **(B)** Quantitation of inflamed areas of the H&E-stained samples of **(A)** is presented as bar graphs. All data for inflamed areas were analyzed by one-way ANOVA followed by Tukey’s multiple comparison test to evaluate significance, and the results are represented as the mean value ± S.D. **p < 0.01, ***p < 0.001 and *n.s.*, not significant. Pre-Tx, pre-treatment; CTL, untreated control; CLR-EMB-RIF, standard regimen; CLR-EMB-CFZ, CFZ-containing regimen.

Histopathological staining showed inflamed areas in *M. avium*-infected lungs (Figure 5A), consistent with the bacterial loads. Surprisingly, the inflamed area was diminished by the CFZ-containing regimen at only 2 weeks after starting treatment (*p* < 0.001) and was reduced even more than at treatment initiation (*p* < 0.001), but there was no significant difference in lung gross pathologic findings between the untreated control group and standard regimen group. At 4 weeks post-treatment, both the standard regimen group and CFZ-containing regimen group had significantly reduced inflamed areas (*p* < 0.001), and treatment with the CFZ-containing regimen resulted in more antibacterial activity than treatment with the standard regimen (*p* < 0.01) (Figure 5B).

## Discussion

Although previous studies showed that a treatment regimen containing CFZ resulted in favorable outcomes for patients with MAC-PD, there have been few studies elucidating its effect on macrophages and relevant animal models. In the present study, CFZ showed effective intracellular anti-MAC activity against all MAC strains, and the CFZ-containing regimen provided significantly improved treatment efficacy in a murine model of chronic progressive MAC-PI, with greater efficacy after 2 weeks of treatment with this regimen than after 4 weeks of treatment with the standard regimen, by rapidly reducing bacterial loads and lung inflammation. Recently, due to a lack of compatible new drugs with low toxicity and high effectiveness, existing drugs have been repurposed or repositioned as the current candidate drugs for the treatment of MAC disease (Wu et al., 2018).

Macrolides have been considered the cornerstone drugs in the treatment of MAC disease, followed by EMB and RIF (Daley et al., 2020). Kim et al. (2019) reported that MAC-PD patients who maintained treatment with either EMB or RIF with a macrolide had a higher probability of culture conversion than patients who maintained treatment with a macrolide only, but maintenance with EMB was superior to maintenance with RIF in terms of treatment outcomes and microbiological cure rates. In addition, Miwa et al. (2014) reported that a double regimen without RIF was not inferior to a standard regimen with respect to culture conversion after 12 months of treatment in patients with MAC. Additionally, clinical research regarding the CFZ-containing regimen and RIF-containing regimen demonstrated that 100% of patients treated with CFZ achieved negative culture results, while only 71% of patients treated with RIF had successful sputum culture conversion (Jarand et al., 2016). For this reason, we investigated the individual efficacy of first-line antibiotics through intracellular activity in MAC-infected BMDMs. The main limitation of *in vitro* DST is that fixed breakpoints of EMB and RIF have not been clearly established, although those drugs have been considered first-line drugs (CLSI, 2018). Therefore, we attempted to evaluate the intracellular anti-MAC activities of these drugs prior to conducting *in vivo* efficacy testing since *in vitro* DST results varied according to MAC strains. To evaluate the intracellular efficacies of antibiotics, seven different MAC strains showing different MIC values against antibiotics were used. Intriguingly, RIF did not confer anti-MAC activity in BMDMs despite its low MIC, regardless of the macrophage source (i.e., different mouse strains). Surprisingly, few studies have investigated the intracellular activity of RIF against MAC infection in macrophages. Moreover, no consistent results among studies have been reported; Rastogi et al. (1987) and Rastogi and Labrousse (1991) showed that RIF was bactericidal for *M. avium* in the J774 mouse macrophage cell line, whereas Yajko et al. (1989) reported that 11 of 13 MAC strains survived in the same J774 cells (with more than 90% of the initial number in infected cells) after treatment with 8 mg/L RIF for 7 days.

The *in vitro* activity of CFZ against the MAC is also still controversial. Huang et al. (2018) reported CFZ MIC ranges from 1 to 4 mg/L for 83 clinical MAC isolates of *M. avium*, with MICs ≤ 1 mg/L against the majority of *M. intracellulare* isolates. In addition, Kwak et al. (2020) reported that the MICs of CFZ for 133 MAC strains ranged from 0.031 to 8 mg/L, and more than 75% of the MAC isolates had MIC values ≤ 1 mg/L. The cut-off values of several studies could be variable due to the use of different MAC species and different culture conditions. In our study, a total of seven MAC strains had MIC values from 1 to 4 mg/L, consistent with the range reported for CFZ in previous studies (Huang et al., 2018; Kwak et al., 2020). Since drug options have been limited for MAC-PD treatment, the general choice of drug regimen has been empirically selected based on MIC determination (Renvoise et al., 2014). However, unexpectedly, RIF showed the weakest correlation between its *in vitro* MIC and intracellular anti-MAC activity. In contrast, in our investigation, CFZ could be an effective drug displaying positive correlations between the *in vitro* MIC and intracellular anti-MAC activity, similar to CLR. In addition, CFZ had better synergistic effects with CLR than with RIF on growth inhibition of all strains except *M. avium* SMC #7 in macrophages. Indeed, many previous studies have reported variation in virulence among MAC strains and different host responses in macrophages and mice (Amaral et al., 2011; Agdestein et al., 2014; Bruffaerts et al., 2017; Abdissa et al., 2018; Rindi et al., 2018; Kannan et al., 2019). Moreover, antibiotics are well known to be associated with immune responses (Labro and Abdelghaffar, 2001; Tamaoki, 2004; Yuhas et al., 2007; Yuhas et al., 2011; Singh et al., 2014). Hence, variation in virulence and host responses and the influence of antibiotics might explain why intracellular *M. avium* SMC #7 was inhibited by both regimens with the same efficacy. Therefore, to evaluate the efficacies of the regimens for chronic progressive disease, mice were infected via aerosol infection with a variety of MAC strains including *M. avium* ATCC 700898 and *M. avium* SMC #7. However, mice infected with various MAC strains and *M. avium* ATCC 700898 underwent bacterial clearance, and chronic progressive infection could not be established (data not shown), whereas mice infected with *M. avium* SMC #7 established a chronic pulmonary infection with granulomatous inflammation. Although we found that the CFZ-containing regimen was superior to the standard regimen through the *in vivo* study with *M. avium* SMC #7, the intracellular activity of CFZ-containing regimen displayed a lesser extent to the other MAC strains in BMDMs. We assume that the difference in results between intracellular activity of CFZ and its *in vivo* efficacy testing against *M. avium* SM #7 might be from the frequencies of treatment and immune status between BMDMs and animals. Thus, the refined intracellular drug assays that mimic *in vivo* conditions should ensure more accurate results, particularly, when testing new drugs. Nevertheless, our results clearly displayed the effective intracellular activity of CFZ against various clinical MAC strains in BMDMs.

Since effective MAC-PD treatment requires at least 12 months of combination therapy (Daley et al., 2020) and since disease progression may worsen radiologically due to severe lung inflammation (Thomson and Yew, 2009), evaluating lung inflammation during antibiotic treatment is necessary to devise more effective MAC-PD chemotherapy regimens. Accordingly, we showed that lung inflammation and bacterial loads declined much earlier in mice treated with the CFZ-containing regimen than in mice treated with the standard regimen. Lung inflammation in mice treated with the CFZ-containing regimen was significantly reduced at 2 weeks post-treatment, whereas lung inflammation in mice treated with the standard regimen was not significantly reduced at this time point. Moreover, treatment with a CFZ-containing regimen at 2 weeks post-treatment was more effective than treatment with a standard regimen for 4 weeks, indicating that the CFZ-containing regimen also had a treatment-shortening effect.

Overall, we evaluated a CFZ-containing regimen for the treatment of a murine model of chronic progressive MAC-PI based on its intracellular activity against a variety of MAC strains in BMDMs. These results provide a rationale for the use of CFZ as a component of an optimal treatment regimen instead of the currently used standard regimen. Furthermore, investigations of treatment regimens combining macrolides and CFZ with other drugs to enhance treatment success in a short duration are needed. Another important finding is that RIF generally had no intracellular anti-MAC activity despite its low MICs against MAC strains, indicating that the host-mediated mechanism of RIF tolerance in macrophages is worth further investigation.

## Data Availability Statement

The original contributions generated for this study are included in the article/Supplementary Material, further inquiries can be directed to the corresponding author/s.

## Ethics Statement

The animal study was reviewed and approved by the Ethics Committee and Institutional Animal Care and Use Committee of the Laboratory Animal Research Center at Yonsei University College of Medicine (Permit Numbers: 2015-0273 and 2018-0229).

## Author Contributions

L-HK and SS conceived, designed, and supervised the work. JL and JP together with L-HK and SC analyzed the data and interpreted the results. BJ and S-YK provided the clinical MAC isolates obtained from MAC-PD patients. JL and JP drafted and prepared the manuscript while BJ, S-YK, K-WJ, JH, L-HK, and SS were involved in the editing of the manuscript. All authors read and approved the final version.

## Conflict of Interest

The authors declare that the research was conducted in the absence of any commercial or financial relationships that could be construed as a potential conflict of interest.
