# Supplementary material for: A Clofazimine-Containing Regimen Confers Improved Treatment Outcomes in Macrophages and in a Murine Model of Chronic Progressive Pulmonary Infection Caused by the Mycobacterium avium Complex
Source: Front Microbiol. 2021 Jan 14;11:626216. doi: 10.3389/fmicb.2020.626216 (PMC7841306; doi:10.3389/fmicb.2020.626216)
Supplement: Supplementary file 1 [file Data_Sheet_1.DOCX]

Supplementary Material

# Supplementary Figures and Tables

## Supplementary Figures


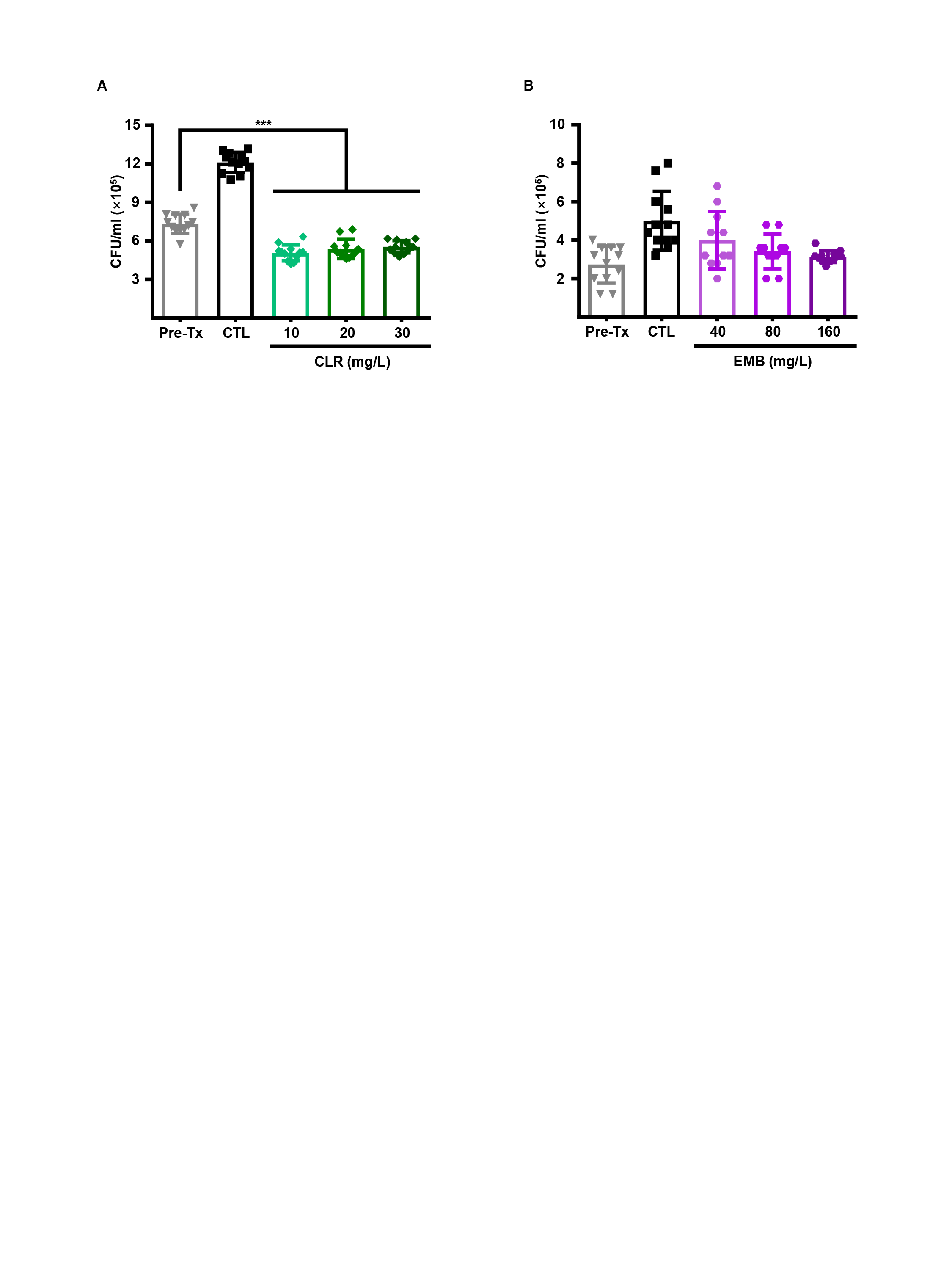


**Supplementary Figure 1.** Dose-dependent intracellular activities of CLR and EMB in MAC-infected BMDMs. BMDMs were infected with *M. avium* ATCC 700898 and treated with the indicated doses of **(A)** CLR and **(B)** EMB. After 72 h of cultivation, bacterial CFUs were enumerated by plating serially diluted cell lysates on 7H10-OADC agar plates. Each experiment was repeated at least twice independently with triplicate wells; the results of a representative experiment are shown. Each dot represents the mean value ± S.D. of duplicate or triplicate wells, with four spots applied per well. The Mann-Whitney test was used to evaluate significance, and the results are represented as the mean value ± S.D. ********p* < 0.001. Pre-Tx, pre-treatment; CTL, untreated control.

**
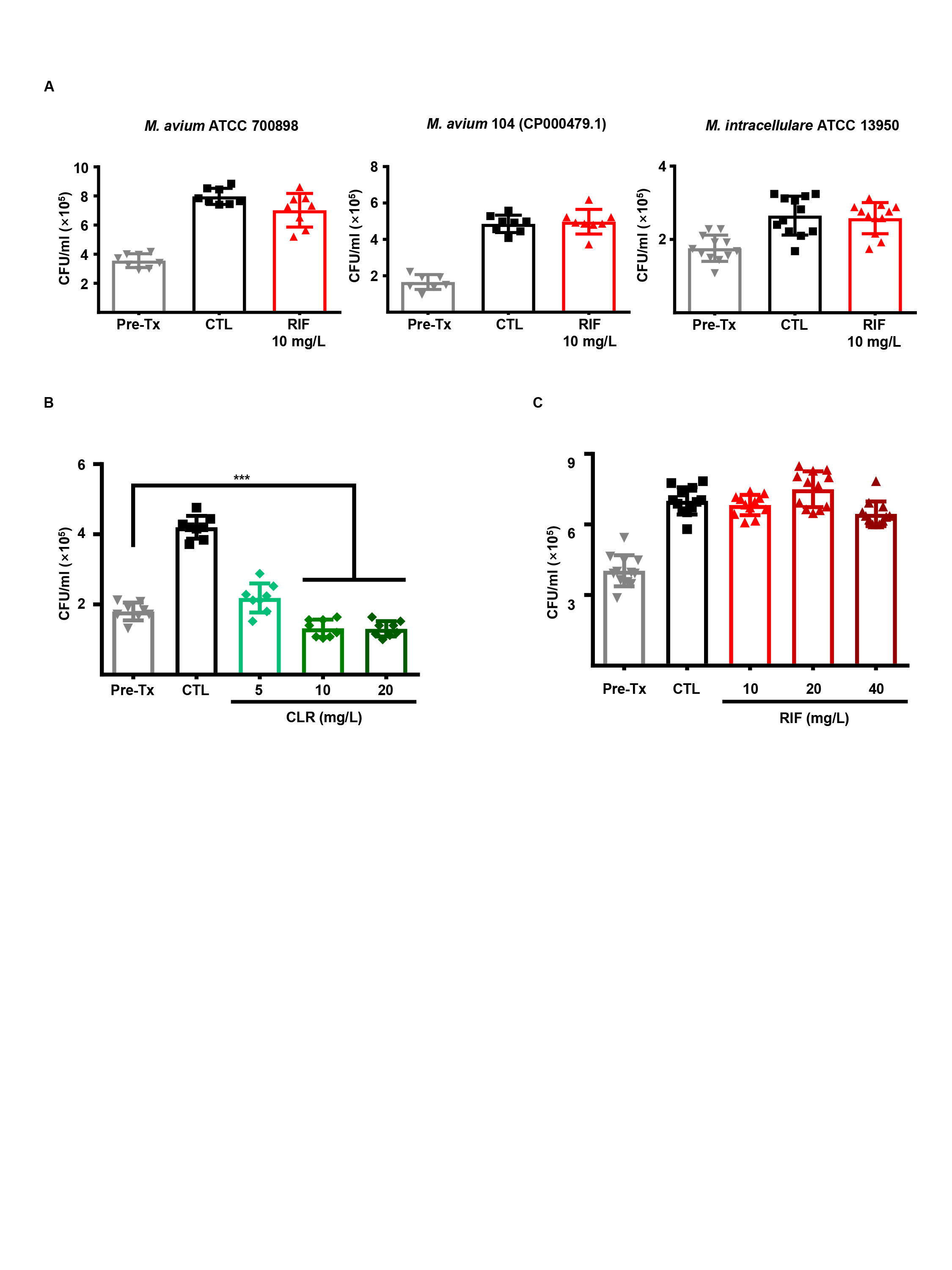
**

**Supplementary Figure 2.** Evaluation of the intracellular activities of first-line drugs in MAC-infected BMDMs from a different murine strain: C57BL/6. **(A)** BMDMs were infected with *M. avium* ATCC 700898, *M. avium* 104 (CP000479.1) or *M. intracellulare* ATCC 13950 and treated with 10 mg/L RIF. BMDMs were infected with *M. avium* 104 (CP000479.1) and treated with the indicated doses of **(B)** CLR and **(C)** RIF. After 72 h of cultivation, bacterial CFUs were enumerated by plating serially diluted cells on 7H10-OADC agar plates. Each experiment was repeated at least twice independently with duplicate or triplicate wells; the results of a representative experiment are shown. Each dot represents the mean value ± S.D. of duplicate or triplicate wells, with four spots applied per well. The Mann-Whitney test was used to evaluate significance, and the results are represented as the mean value ± S.D. ********p* < 0.001. Pre-Tx, pre-treatment; CTL, untreated control.

**
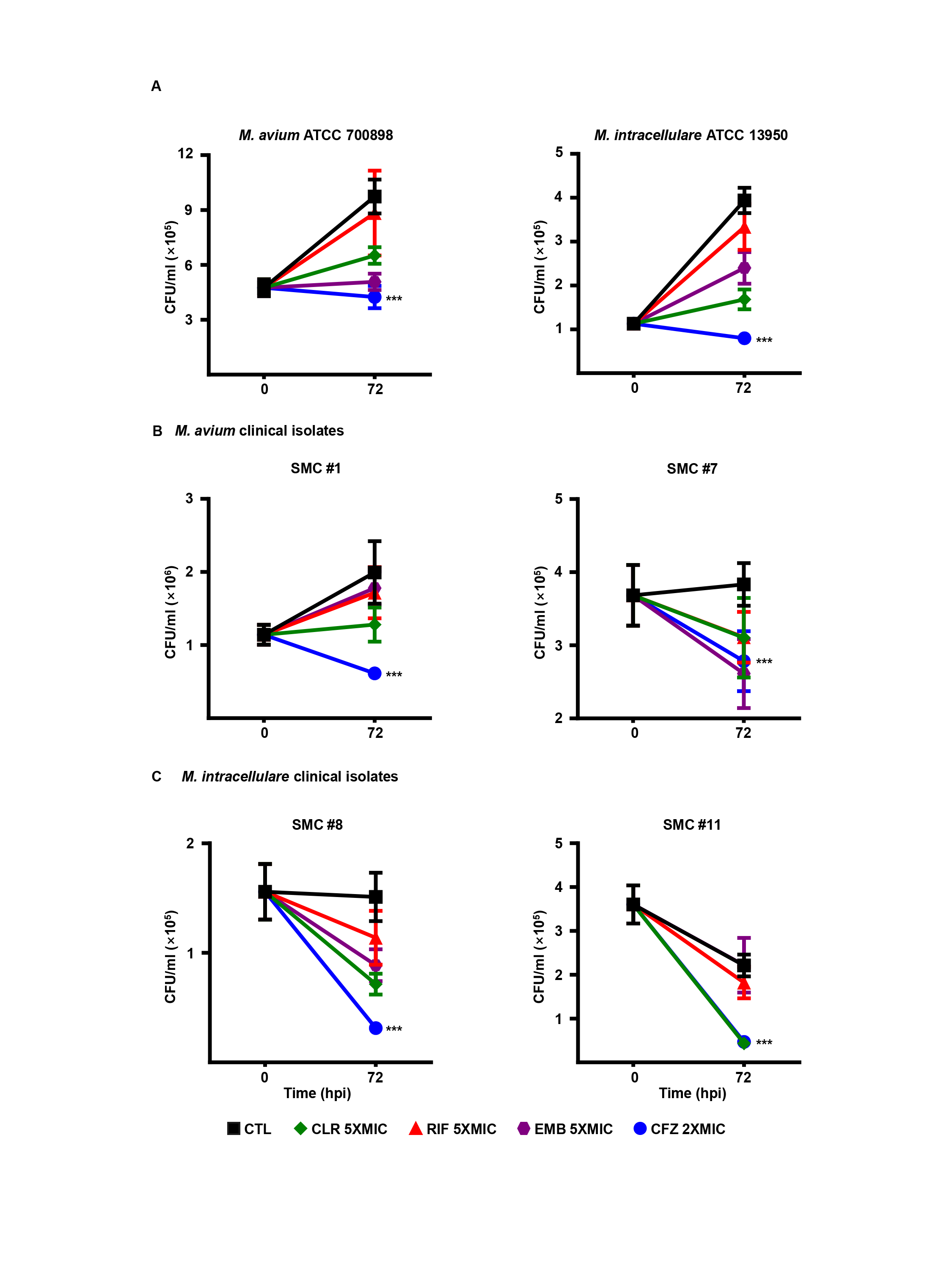
**

**Supplementary Figure 3.** Comparative evaluation of the intracellular activities of individual drugs (CLR, EMB, RIF, and CFZ) against a variety of MAC strains in BMDMs. BMDMs were infected with each MAC strain and treated with the indicated multiples of the MIC according to the data in Table 1. **(A)** *M. avium* ATCC 700898 and *M. intracellulare* ATCC 13950, **(B)** *M. avium* SMC #1 and *M. avium* SMC #7 and **(C)** *M. intracellulare* SMC #8 and *M. intracellulare* SMC #11 were assessed at 72 h post-infection by plating serially diluted cell lysates on 7H10-OADC agar plates. Each experiment was repeated at least twice independently with triplicate wells; the results of a representative experiment are shown. The Mann-Whitney test was used to evaluate significance, and the results are represented as the mean value ± S.D. ********p* < 0.001 vs. 0 hpi. CTL, untreated control; hpi, h post-infection.

**
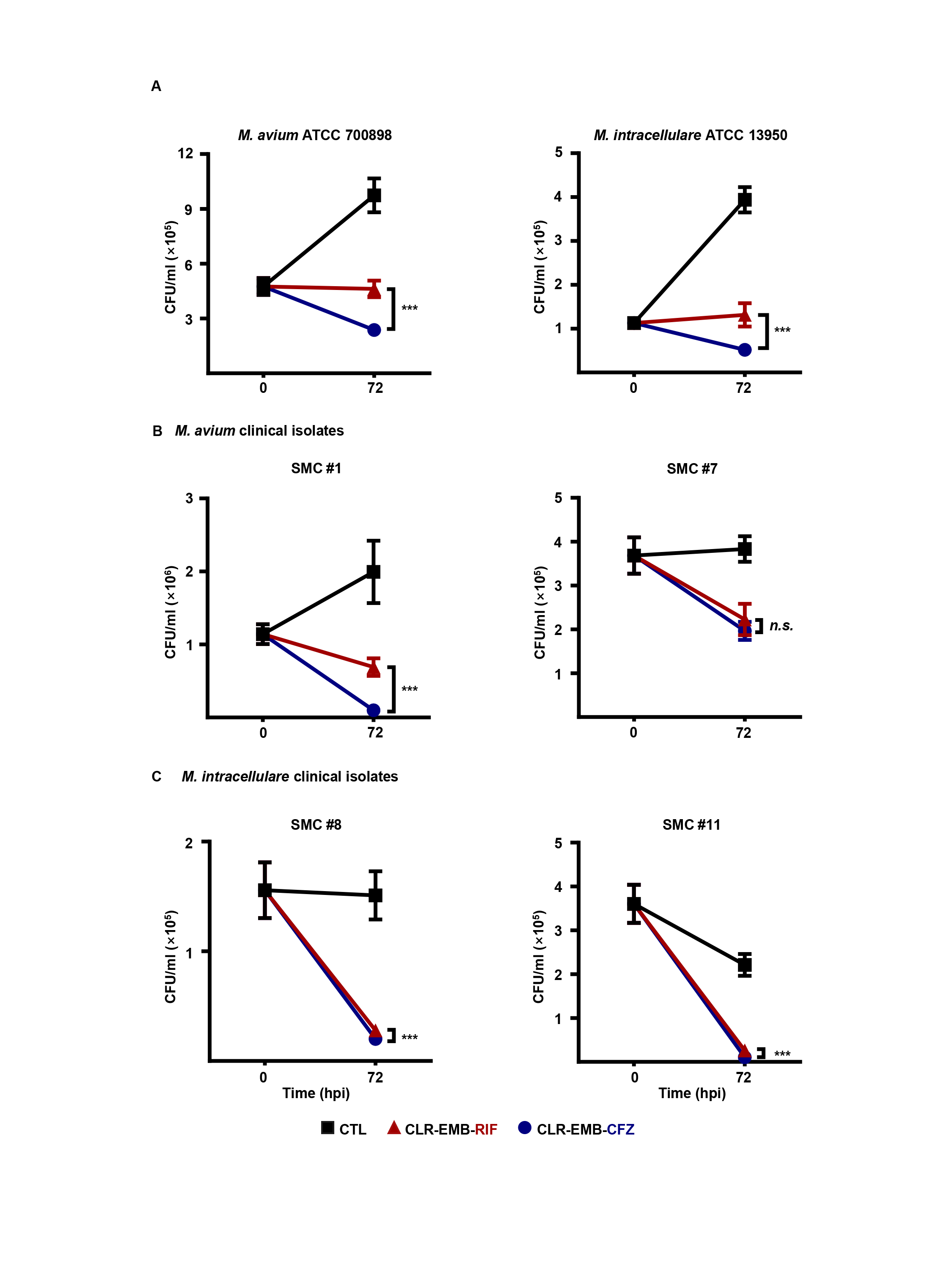
**

**Supplementary Figure 4.** Comparative evaluation of the intracellular activities of drug combinations for the standard regimen and for the CFZ-containing regimen against a variety of MAC strains in BMDMs. BMDMs were infected with each MAC strain and treated with 3×MIC according to the data in Table 1. **(A)** *M. avium* ATCC 700898 and *M. intracellulare* ATCC 13950, **(B)** *M. avium* SMC #1 and *M. avium* SMC #7 and **(C)** *M. intracellulare* SMC #8 and *M. intracellulare* SMC #11 were assessed at 72 h post-infection by plating serially diluted cell lysates on 7H10-OADC agar plates. Each experiment was repeated at least twice independently with triplicate wells; the results of a representative experiment are shown. The Mann-Whitney test was used to evaluate significance, and the results are represented as the mean value ± S.D. ********p* < 0.001 and *n.s.*: not significant. CTL, untreated control; CLR-EMB-RIF, standard regimen; CLR-EMB-CFZ, CFZ-containing regimen; hpi, h post-infection.
